# Conformance-Aware Predictive Process Monitoring for Early Detection of Sepsis Deterioration Using Incomplete Care Pathways

**DOI:** 10.3390/jcm15051956

**Published:** 2026-03-04

**Authors:** Kimberly D. Harry, Mohammad Najeh Samara

**Affiliations:** School of Systems Science and Industrial Engineering, Thomas J. Watson College of Engineering and Applied Science, Binghamton University, Binghamton, NY 13902, USA; msamara1@binghamton.edu

**Keywords:** process mining, machine learning, healthcare, sepsis, early detection, care pathway

## Abstract

**Background/Objectives**: Sepsis is a leading cause of morbidity and mortality due to its rapid progression and variability in care delivery. While existing predictive models estimate sepsis risk using clinical variables, they typically rely on static attributes and overlook temporal, behavioral, and process-related characteristics of care pathways. In particular, deviations from recommended protocols and process inefficiencies are rarely incorporated into early deterioration prediction. This study proposes a Conformance-Aware Predictive Process Monitoring (CAPPM) framework to enable early detection of sepsis deterioration using incomplete care pathways. **Methods**: The proposed framework integrates process mining with predictive modeling. Using the publicly available Sepsis Cases Event Log, we first discovered the reference care pathway and generated prefix-level representations of ongoing cases. Temporal and behavioral features were engineered alongside alignment-based and declarative conformance metrics to quantify pathway deviations. These features were used to train and evaluate multiple supervised learning models, including Adaptive Boosting and Gradient Boosting. Predictive performance was assessed using the area under the receiver operating characteristic curve (AUROC). **Results**: Incorporating conformance and pathway-based features improved predictive performance compared to models relying solely on traditional attributes. Adaptive Boosting and Gradient Boosting achieved the strongest results, with AUROC values of 0.744 and 0.731, respectively, demonstrating enhanced early detection ability. **Conclusions**: The findings indicate that early deviations in care pathways and temporal progression patterns provide meaningful predictive signals for sepsis deterioration. Integrating process mining with machine learning offers a promising approach for time-critical clinical decision support and proactive intervention.

## 1. Introduction

Sepsis, a life-threatening organ dysfunction caused by a dysregulated host response to infection, remains one of the most critical challenges in modern healthcare [1]. According to the Global Burden of Diseases, Injuries, and Risk Factors Study (GBD) in 2021, there were approximately 166 million sepsis-related cases and 21.4 million sepsis-related deaths worldwide, representing nearly 31.5% of all global deaths [2]. Early identification and timely intervention are crucial to prevent sepsis condition deterioration. However, the inherent complexity of healthcare systems and numerous interrelated processes often lead to deviations from the optimal care pathways, resulting in inefficiencies, delayed interventions, and rapid patient deterioration.

While data-driven approaches using artificial intelligence (AI) have been widely employed to predict sepsis risk [3,4,5], they treat clinical data or event logs as static instances rather than a dynamic temporal sequence of events that occur over time. To address this challenge, Process Mining (PM) has emerged as a powerful discipline used to discover, monitor, analyze, and visualize real-world processes by extracting valuable insights from event logs and identifying bottlenecks [6,7]. However, traditional PM techniques are predominantly retrospective, analyzing completed workflows after execution, which in healthcare typically correspond to patient trajectories from admission to a terminal outcome such as discharge or death [8,9].

Recent advancements in Predictive Process Monitoring (PPM) attempt to bridge this gap by predicting the future of ongoing cases [8,10]. However, two critical limitations exist in the current literature. First, most PPM techniques rely on complete or near-complete traces, which are clinically unrealistic for sepsis, where decisions must be made in the first few hours. Second, existing models largely ignore conformance features, specifically, whether a patient’s current trajectory violates established medical protocols. This is a critical oversight, as non-conformance, for example, delayed antibiotics, is often a precursor to septic shock [11].

To address these gaps, this paper proposes a novel Conformance-Aware Predictive Process Monitoring (CAPPM) framework for early detection of sepsis deterioration using incomplete care pathways and combining AI/ML and PM techniques. The proposed framework integrates temporal, behavioral, and conformance-based features extracted from prefix-based traces to capture the progression of care and detect deviations from reference clinical models. By monitoring incomplete prefixes against a reference clinical model, we can detect deterioration risks that stem from workflow failures, not just physiological changes.

The key contributions of this paper are as follows:

- A conformance-aware predictive monitoring framework that supports early detection of sepsis deterioration from incomplete care pathways.
- A method for incorporating alignment-based conformance information, including prefix-level costs and trend indicators, into predictive modelling capability.
- A pathway profiling approach that captures structural differences in patient trajectories and enhances early sepsis risk identification.
- A unified feature representation that combines clinical, temporal, behavioral, and conformance signals to improve predictive accuracy.

The rest of the paper is organized as follows: Section 2 presents a review of existing works on early sepsis prediction and detection using AI/ML, as well as the integration of PM. The methodology adopted for this study is presented and discussed in Section 3. Section 4 presents and discusses the results of the analysis, and Section 5 provides the limitations of the study and presents recommendations for future research. Finally, Section 6 concludes the paper.

## 2. Literature Review

This section reviews the literature on early sepsis detection, covering the application of AI/ML models and the evolution of process mining in healthcare. The review focuses particularly on the recent integration of these techniques to achieve predictive process monitoring.

The literature review was conducted to identify recent advances in sepsis prediction, process mining in healthcare, and predictive process monitoring. Publications were retrieved using databases including PubMed, Scopus, IEEE Xplore, and Google Scholar. Searches were performed using combinations of keywords such as sepsis prediction, process mining in healthcare, predictive process monitoring, care pathway analysis, and machine learning for clinical deterioration. Priority was given to peer-reviewed journal and conference papers published primarily between 2015 and 2025, with earlier foundational works included where necessary. Studies were selected based on relevance to sepsis management, healthcare workflow analysis, or predictive process monitoring, with emphasis on works integrating process mining and predictive analytics.

### 2.1. AI/Machine Learning for Sepsis Detection

Sepsis is a life-threatening medical condition triggered by the body’s dysregulated immune response to infection, leading to tissue damage, organ dysfunction, and potentially death if not promptly treated [12]. It remains one of the leading causes of mortality and morbidity globally, particularly among critically ill and hospitalized patients. Due to its rapid progression and highly heterogeneous clinical presentation, early detection of sepsis is essential for improving patient outcomes. Recent advances in AI and ML have enabled significant progress in early prediction, diagnosis, and mortality risk assessment for sepsis [3,13,14]. Predication models based on deep learning, gradient boosting, random forests, and ensemble methods have demonstrated strong predictive performance using electronic health record (EHR) data, vital signs, laboratory results, and clinical notes.

However, despite their predictive accuracy, these models treat healthcare data as static and fail to consider the temporal, sequential, and process-oriented nature of clinical care. In real-world sepsis management, patient care unfolds through complex clinical workflows involving multiple healthcare professionals, diagnostic procedures, interventions, and time-sensitive decisions, elements that are not captured by conventional ML predictions. To address this limitation, process mining has emerged as a complementary approach that focuses on modeling and analyzing actual care pathways using event logs [15]. It enables the extraction and visualization of real-world healthcare workflows, including treatment deviations, bottlenecks, delays, and compliance with medical guidelines. Through process discovery, conformance checking, and enhancement, it provides actionable insights into operational efficiency, quality of care, and protocol adherence [16]. Several extensive reviews have been conducted exploring its applications as well as challenges in the healthcare system, providing valuable trends, insights, and directions for further research [17,18,19,20].

### 2.2. Process Mining and Predictive Integration

Exploring the applicability to sepsis-related clinical and operational contexts [21,22], studies conducted PM on hospital event logs of emergency department patients with sepsis to map real-world clinical workflows, identify bottlenecks, and uncover potential inefficiencies in sepsis management. These studies demonstrated that there are possible associations between discharge methods and patient readmissions, emphasizing the value of process-aware analysis for improving operational efficiency and clinical outcomes. Bakhshi et al. [23] applied Heuristics Miner (HM) and Inductive Miner (IM) to real-world sepsis patient trajectories and demonstrated that they struggle to model complex clinical pathways accurately. Similarly, authors [24] applied Alpha Miner, Heuristic Mining, and Direct Flow Graph (DFG) to a specific sepsis treatment dataset, where it was shown that PM can uncover deviations and bottlenecks in treatment workflows, offering valuable insights for improving care quality, reducing costs, and optimizing sepsis management processes.

Process Mining Project Methodology in Healthcare (PM^2^HC) was employed in [25] to analyze and compare sepsis treatment workflows across different patient subpopulations, such as severe and non-severe cases. Results obtained showed that distinct patient groups follow substantially different treatment trajectories and exhibit varying outcomes, emphasizing the importance of subgroup-specific workflow analysis to enable more tailored, efficient, and outcome-oriented sepsis management. Real-world trajectories of sepsis patients from emergency admission to discharge were analyzed in [26,27] using PM to discover workflow models, assess conformance with clinical guidelines, and visualize care flows. Results showed that PM can clarify patient flows, reveal deviations from recommended protocols, and uncover challenges in data quality and model interpretability, highlighting its value as an iterative, insightful tool for analyzing sepsis care processes.

These research works have demonstrated that PM can map/visualize patients’ workflow, uncover inefficiencies, reveal deviations, and remove bottlenecks, particularly considering the sepsis condition. However, it remains retrospective and descriptive, limiting its use in real-time clinical decision support. To address this limitation, recent works integrate ML with PM. For example, A previous study [28] applied the DFG process mining technique to the MIMIC-IV-ED dataset to extract emergency care pathways, pre-processed them into structured event-based features, and evaluated seven tree-based and boosting ML classification algorithms to predict clinical and operational outcomes. Results showed that RF and Extreme Gradient Boosting (XGBoost) consistently performed better than the other models across all metrics. Similarly, the authors in [29] combined PM with ML on the MOVER EHR dataset to model real-world surgical workflows, detect bottlenecks, and generate predictive insights related to operating room efficiency and postoperative outcomes. A multi-stage framework was implemented, including heuristic control-flow discovery, Petri net-based conformance checking, temporal performance analysis, unsupervised clustering, and RF-based classification, where it was seen that the process model that achieved perfect fitness of 1.0 and moderate precision of 0.46, while RF model achieved 90.33% accuracy but only 24.23% recall for delayed recovery cases, highlighting challenges in detecting high-risk patients. ML was also utilized in [30] to improve the clinical process in sepsis management. While the research work did not conduct simulations or analysis, it provided valuable insights into the benefits as well as the drawbacks of leveraging ML for process improvement.

**Figure 1:**
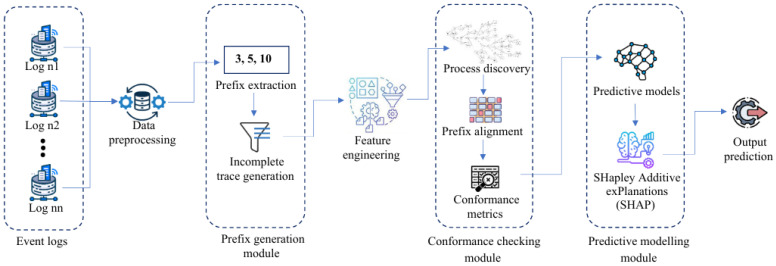
CAPPM Framework Architecture.

From the review, it can be seen that while ML models provide good predictive accuracy, they largely ignore the temporal and process-oriented nature of sepsis care, and existing PM applications, although useful for analyzing workflows and detecting deviations, remain retrospective and lack predictive capabilities. Few studies have attempted to integrate PM with ML, and those that did focused mainly on surgical or operational settings, with limited attention to early sepsis deterioration and little use of conformance metrics as predictive features. This highlights a clear research gap, which this study addresses by proposing a Conformance-Aware Predictive Process Monitoring (CAPPM) framework that leverages incomplete care pathways to enable early detection of sepsis deterioration. To clarify conceptual differences between traditional PM, Predictive PM, and the proposed CAPPM framework, Table 1 summarizes their primary characteristics and intended use. Figure 1 below illustrates a graphic showing the SEPSIS care pathway predictive process integrating behavioral features, temporal features, clinical features, conformance-based features, and care pathway features (i.e., viz, sepsis type, and protocol/treatment stages).

## 3. Research Methodology

This section presents the methodology adopted for this research, comprising the dataset description, the data preprocessing steps, and the proposed CAPPM framework. The framework is designed to integrate PM capabilities with ML to enable early detection of sepsis deterioration.

All process mining analyses were conducted using PM4Py (Process Mining for Python) version 2.7.11 (Eindhoven University of Technology, Eindhoven, The Netherlands). Machine learning models were implemented in Python version 3.12.2 (Python Software Foundation, Wilmington, DE, USA) using scikit-learn version 1.4.2 (scikit-learn developers, online resource). SHAP analysis was performed using the SHAP library version 0.45.0 (online resource). All computations were executed in a standard Python environment.

### 3.1. Dataset Description

This study utilizes the publicly available Sepsis Cases Event Log originally published by Mannhardt [31], which contains real-world patient trajectories from a Dutch academic hospital. The event log records the sequence of activities from Emergency Room (ER) registration until discharge and covers approximately one year of hospital operations. Each trace represents the treatment pathway of a single patient, recorded in eXtensible Event Stream (XES) format.

Each event is at least annotated with a

- a case identifier (*case:concept:name*),
- an activity label (*concept:name*),
- a timestamp (*time:timestamp*).

The main clinical activities include ER Registration, ER Triage, ER Sepsis Triage, various laboratory tests, including C-reactive protein (CRP), Leucocytes, and LacticAcid, administration of IV Liquid and IV Antibiotics, ward and intensive care admissions include Admission NC and Admission IC, possible returns to the ER, and several discharge or release outcomes. Table 2 presents descriptive statistics of activity occurrences across all cases. The event log also contains numeric clinical values for selected laboratory tests that are used as time-varying attributes in the predictive models. The event log provides a moderate number of patient cases and events, which is suitable for process discovery and predictive modelling while still reflecting the complexity of real sepsis care.

### 3.2. Data Preprocessing

The data processing pipeline focused on turning the raw sepsis event log into a structured, temporally consistent event log suitable for process discovery, conformance checking, and predictive modelling. It followed a stepwise methodology discussed as follows.

#### 3.2.1. Import and Standardization

The original XES file is imported using the PM4Py toolkit, and the columns are renamed to the standard identifiers, timestamps are converted to a uniform time zone, and events are sorted by time within each case. Before training, missing numerical values were imputed using median imputation to ensure model compatibility. This generates a clean event log, mathematically represented as:

(1) $$L=\left\{\tau_{1},\tau_{2},\ldots ,\;\tau_{N}\right\},$$

where each trace $\tau_{1}=\left(e_{i1},\ldots ,e_{in_{i}}\right)$ is a time-ordered list of events.

#### 3.2.2. Outcome Labelling

To support supervised learning, every case is labelled as either deteriorated or non-deteriorated, such that

(2) D={Admission IC, Release D}

is the set of deterioration-related activities. A binary outcome $y_{i}$ is defined for each trace $\tau_{1}$ as

(3) $$y_{i}=\left\{\begin{array}{l} 1,\;if\;∋\;j\;such\;that\;a_{ij}\in D,\;\\ 0,\;otherwise, \end{array}\right.$$

where $a_{ij}$ denotes the activity of events $e_{ij}$. These events indicate transfer to an intensive care unit or discharge outcomes that are associated with more severe clinical status, including death. The same label is applied to all prefixes derived from the original case.

In this study, deterioration is operationally defined using IC admission and severe discharge outcomes recorded in the event log, as these events reliably indicate clinical worsening within the available dataset. Alternative definitions based on delayed IC admission, mortality, or shock-related indicators may further refine deterioration labeling. However, such variables are not consistently available in the current dataset.

### 3.3. CAPPM Framework

The architecture of the proposed CAPPM framework is presented in Figure 1. It consists of sequential modules designed to extract process knowledge and integrate it into a predictive workflow. The framework consists of four sequential stages: (1) Data Preprocessing and Prefix Generation, where raw event logs are cleaned and truncated into incomplete care pathways prior to any deterioration event; (2) Feature Engineering, which extracts behavioral, temporal, and clinical attributes from the incomplete traces; (3) Conformance Checking, which aligns the prefixes against a discovered reference Petri net to compute deviation metrics and pathway clusters; and (4) Predictive Modelling, where machine learning classifiers predict deterioration risk. Feature contributions are interpreted using SHapley Additive exPlanations (SHAP), a method that quantifies the impact of each feature on the model’s prediction.

#### 3.3.1. Prefix Generation

The key objective of this study is to predict early sepsis deterioration from incomplete care pathways. Based on this objective, the goal is to simulate a real-time clinical environment where the final patient outcome is unknown and where early detection of sepsis deterioration is accomplished. The CAPPM framework utilized a prefix-based approach. Rather than analyzing completed traces, this module generates partial traces of fixed lengths k∈{3, 5, 10}, which correspond roughly to early, intermediate, and more advanced stages of the care trajectory. Each prefix keeps the original case identifier and is tagged by its length. This yields a prefix log that contains multiple partial views of the same patient at different stages of the care pathway.

To ensure that predictions simulate a realistic early-warning scenario and avoid information leakage, prefixes containing deterioration-defining events, such as Admission IC or severe discharge outcomes, were excluded from prediction evaluation. Only prefixes occurring strictly before the first deterioration-related event were retained for training and evaluation, ensuring that the model predicts deterioration risk prior to its occurrence. Train–test splitting was performed at the case level rather than at the prefix level. All prefixes derived from a given patient case were assigned exclusively to either the training or testing set. Mathematically, for each prefix observed at time t, the predictive task estimates the conditional probability:

(4) P(Y=1∣events observed up to time t)

where Y=1 indicates that deterioration will occur at any future time $t^{\prime }>t$. This formulation ensures a strict temporal ordering between observed features and the predicted outcome. The predictive task does not rely on a fixed time horizon. Instead, for each prefix observed at time t, the model estimates the cumulative probability that deterioration will occur at any future time point after  t. This formulation aligns with continuous clinical monitoring, where risk is dynamically updated as new events occur, rather than predicting deterioration within a predefined number of hours.

#### 3.3.2. Feature Engineering

The CAPPM framework combines several groups of features at the prefix level so that they reflect the information available at prediction time. These include behavioral, temporal, clinical, and conformance-based and pathway features.

**A. Behavioral Features**

Behavioral features describe how many and which activities have occurred so far. Specifically, it captures the execution flow, including the number of unique activities, the occurrence of specific events. For each prefix, the following quantities are computed:

- number of events n
- number of distinct activities $\left|\left\{a_{i1},\ldots ,\;a_{in}\right\}\right|$
- revisit ratio R

(5) $$R_{i}^{n}=1-\frac{{|\left\{a_{i1},\ldots ,\;a_{in}\right\}}_{unique}|}{n}$$

Binary indicators capture whether key steps have occurred, such as Admission IC, any ward admission, or Return ER. These features summarize the structural progression of the care pathway up to the current point.

**B. Temporal Features**

Temporal features encode the timing of the prefix. These features quantify the speed of care, including the total elapsed time since registration, the mean time between events, and the time since the last recorded activity. For the event times, $t_{i1},\ldots ,t_{ik}$, the following are calculated:

- elapsed hours (h) since the first event

(6) $$h_{i}^{k}=\frac{t_{ik}-t_{i1}}{3600},$$

- mean inter-event time in hours,
- time since the last event, which can highlight periods of inactivity that may indicate waiting or delays.

**C. Clinical Features**

Clinical features are derived from the numeric laboratory attributes identified during preprocessing. These include the actual values of laboratory tests, for example, LacticAcid and Leucocytes, recorded within the prefix. The framework aggregates these by calculating the mean, minimum, maximum, and last-observed values.

Static case attributes, if available, are added unchanged to all prefixes belonging to that case. This creates a set of baseline features that approximate conventional ML inputs and serve as the comparative baseline in later experiments.

**D. Conformance-Based and Pathway Features**

The feature engineering module also integrates outputs from the conformance module and from pathway clustering. For pathway clustering, each case trajectory was represented using structural characteristics derived exclusively from events occurring prior to the first deterioration-defining event for positive cases. For non-deterioration cases, structural representations were derived from equivalent prefix-based truncations to maintain temporal consistency. Clustering was therefore performed without incorporating post-deterioration information. Feature vectors included trace length, activity presence indicators, and selected activity counts computed only from available prefix information. After standardization, K-means clustering with three clusters was applied in an unsupervised manner without using outcome labels. The resulting cluster assignment was then included as a categorical feature for all prefixes of the corresponding case.

The CAPPM framework is flexible with respect to additional clinical information sources. New diagnostic events, laboratory tests, or rapid pathogen identification technologies can be incorporated as additional activities or attributes within the event log. Corresponding behavioral, temporal, or clinical features can then be extracted without modifying the overall framework. As richer data become available, predictive performance and interpretability may improve through enhanced representation of patient care progression.

#### 3.3.3. Conformance Checking Module

The conformance checking module is central to the CAPPM framework. It quantifies the degree to which each prefix conforms to a reference process model [32]. The module generates the core predictive features that distinguish standard variation from critical workflow failure by quantifying the discrepancy between the current activity and the expected activity. It operates through three distinct stages, which include the process discovery and reference model, prefix alignment calculation, and conformance metrics [32,33].

**A. Process Discovery and Reference Model**

To capture the normative sepsis pathway, the Inductive Miner algorithm was applied to the complete event log. The miner discovers a process tree that is then converted to a Petri net. The Petri net consists of places, transitions that correspond to activities, and flow relations. Initial and final markings define the start and end of a valid process instance [32]. This model serves as a reference for conformance checking. The Petri net provides a compact control-flow description that supports synchronous and asynchronous behavior, choice, parallelism, and loops; these control-flow constructs reflect typical clinical variation, such as parallel ordering of diagnostics and iterative monitoring steps [32].

**B. Prefix Alignment Calculation**

For every generated prefix, an optimal alignment is computed against the reference Petri net. The alignment algorithm finds a minimum-cost mapping between the observed activity sequence and the model transitions by allowing synchronous moves, which pair a matching activity with a model transition. The model-only moves are model transitions executed without a matching log event, while the log-only moves are the logged event without a matching model transition. Each alignment returns an overall cost and the counts of the three move types. Because alignment counts are typically heavy-tailed, raw counts were transformed before using them as learning inputs. Specifically, the natural log plus one transform was applied to all costs to reduce skew, improve numerical stability, and improve performance in tree and boosting models.

(7) $$\log1p\left(x\right)=\log\left(1+x\right)$$

**C. Conformance Metrics**

For each alignment, we derive the following conformance metrics for the prefix-level feature set:

- Alignment cost: the total numeric cost returned by the alignment algorithm. Higher values indicate larger deviations from the reference model. Both the raw cost for inspection and log1p(cost) for modeling.
- Synchronous moves: the number of steps the log and model agree on. Synchrony is indicative of conformity.
- Model moves: model-only moves that indicate expected steps missing from the observed prefix.
- Log moves: log-only moves that represent unexpected activities not permitted by the model.

In addition to these feature sets, trend features across prefixes were also computed for the same case to capture dynamic conformance behavior, and a set of declarative response-violation ratios was computed for selected activation-response pairs. For instance, ER registration to ER triage. For an activation a and a response b, the response violation ratio is the fraction of a-occurrences in the prefix that are not followed by any b later in the same prefix. This value ranges from 0 (no violations or no activations) to 1 (all activations violate the response).

#### 3.3.4. Predictive Model

To achieve predictive monitoring for the early sepsis deterioration within the CAPPM framework, several supervised ML models were implemented and evaluated. Specifically, tree-based and ensemble models were considered as they are well-suited for structured, event-derived data and can effectively capture interactions across the diverse feature groups generated by the CAPPM pipeline. All models were trained using the prefix-level feature matrices generated from the process, conformance, declarative, and clinical components. The aim was to identify the model that provides the most reliable and accurate predictions of sepsis deterioration risk at different stages of the patient care management pathway.

**A. Decision Tree (DT)**

A decision tree is a supervised learning model that organizes decisions in a hierarchical, tree-like structure [28]. At each internal node, the model applies a rule that splits the data into smaller groups based on feature values. This splitting continues until the tree reaches its terminal nodes, where final class predictions are made. Decision trees are intuitive and easy to interpret, but their structure can become overly complex when trained on real-world data, which may lead to reduced generalization if not properly managed.

**B. Random Forest (RF)**

Random Forest extends the idea of a single decision tree by building many trees and combining their outputs [28]. Each tree is trained using a random selection of both samples and features, which introduces variability and reduces the chance of overfitting to the training data. The final prediction is obtained by aggregating the predictions of all trees in the ensemble. This approach improves stability, increases predictive accuracy, and makes Random Forest a strong choice for handling diverse and noisy datasets.

**C. Gradient Boosting (GB)**

Gradient Boosting is an ensemble learning technique that builds predictive models in a sequential manner [28]. Each tree in the sequence attempts to correct the errors of the previous one by focusing on instances that were previously misclassified. This additive approach allows the model to capture complex patterns in the data while maintaining control over overfitting through learning rate adjustments and shallow tree depth. Gradient Boosting is widely applied in predictive analytics due to its strong performance on structured datasets.

**D. Extra Trees (ET)**

The Extra Trees model, also known as Extremely Randomized Trees, is another ensemble of decision trees but introduces additional randomness during the split selection process [28]. Unlike Random Forest, which searches for the best split among a random subset of features, Extra Trees selects split thresholds at random. This increased randomness reduces variance and training time while still maintaining strong predictive performance. Extra Trees often performs well when the dataset contains many correlated or noisy features.

**E. Adaptive Boosting (AdaBoost)**

AdaBoost constructs an ensemble of weak learners where each subsequent learner focuses more heavily on samples that were previously misclassified [28]. By iteratively adjusting the weights of training instances, the algorithm encourages the ensemble to correct earlier mistakes. This results in a strong final classifier formed by a weighted combination of weak learners. AdaBoost is effective for handling imbalanced datasets and can improve performance even with simple base models such as shallow decision trees.

**F. Model Interpretability Using SHAP**

To improve the interpretability of the predictive models, SHAP analysis was applied to quantify how individual features contribute to deterioration predictions. SHAP assigns contribution values to each feature based on cooperative game theory principles, allowing identification of features that increase or decrease predicted risk. This analysis enables interpretation of model outputs in terms of clinical workflow characteristics and supports understanding of how pathway deviations and temporal factors influence predictions.

Although the CAPPM framework is demonstrated using a single hospital dataset, its core components are transferable across institutions. Prefix generation, conformance checking, and predictive modeling procedures can be applied to other healthcare environments. However, the reference process model and pathway structures must be derived from local event logs, as they depend on institution-specific workflows and documentation practices. Implementation in new settings, therefore, requires recalibration using local process data.

### 3.4. Evaluation Metrics

Several metrics were utilized to assess the effectiveness of the predictive models. These metrics were considered because they provide complementary views of model performance, especially in a clinical prediction setting where class imbalance and decision reliability are important.

**A. Area Under the ROC Curve (AUROC)**

The AUROC measures how well a model can distinguish between deterioration and non-deterioration cases across all possible classification thresholds. It is a widely used performance metric for binary classifiers and summarizes the trade-off between the True Positive Rate (TPR) and the False Positive Rate (FPR) across all thresholds [34,35]. A higher AUROC means the model is better at ranking deteriorating cases higher than non-deteriorating ones.

(8) $$TPR=\frac{True\;Positives}{True\;Positives+False\;Negatives}$$

(9) $$FPR=\frac{False\;Positives}{False\;Positives+True\;Negatives}$$

The AUROC value ranges from 0.5 (no discrimination) to 1.0 (perfect discrimination).

**B. Area Under the Precision-Recall Curve (AUPRC)**

AUPRC summarizes the relationship between Precision and Recall across different thresholds. It is especially useful when the positive class (deterioration) is rare. AUPRC focuses specifically on how well the model identifies positive cases, making it more informative than AUROC when the dataset is imbalanced [36]. A higher AUPRC means the model maintains strong precision while still identifying most deterioration cases.

(10) $$Precision=\frac{True\;Positives}{True\;Positives+False\;Positives}$$

(11) $$Recall=\frac{True\;Positives}{True\;Positives+False\;Negatives}$$

**C. Confusion Matrix**

A confusion matrix summarizes how many predictions the model classified correctly or incorrectly [37]. It provides a direct, interpretable view of model errors and is useful for assessing clinical risk, for example, the cost of false negatives. It shows:

- True Positives (TP): deterioration correctly predicted
- False Positives (FP): non-deterioration predicted as deterioration
- True Negatives (TN): non-deterioration correctly predicted
- False Negatives (FN): deterioration missed

**D. Brier Score and Calibration Assessment**

The Brier score measures the mean squared difference between predicted probabilities and observed binary outcomes. For predicted probability $p_{i}$ and true outcome $y_{i}$, the Brier score is expressed mathematically as follows:

(12) $$\mathrm{Brier}=\frac{1}{N}\sum_{i=1}^{N}(p_{i}-y_{i}{)}^{2}$$

Lower Brier scores indicate more accurate and better calibrated probability estimates. Unlike AUROC and AUPRC, which evaluate ranking performance, the Brier score assesses the overall accuracy of predicted risk magnitudes.

Calibration curves group predicted probabilities into bins and compare the mean predicted probability within each bin to the observed event frequency. A perfectly calibrated model would lie on the diagonal line, indicating agreement between predicted risk and actual outcome frequency.

Reliability diagrams provide a graphical representation of this relationship by plotting observed outcome proportions against predicted probabilities across risk strata. These analyses are particularly important in clinical applications, where predicted probabilities may inform decision thresholds and resource allocation.

## 4. Results and Discussion

This section presents and discusses the results obtained from the implementation and evaluation of the proposed CAPPM framework for the early prediction of sepsis deterioration using incomplete care pathways.

### 4.1. Sepsis Care Pathway Discovery

**Figure 2:**
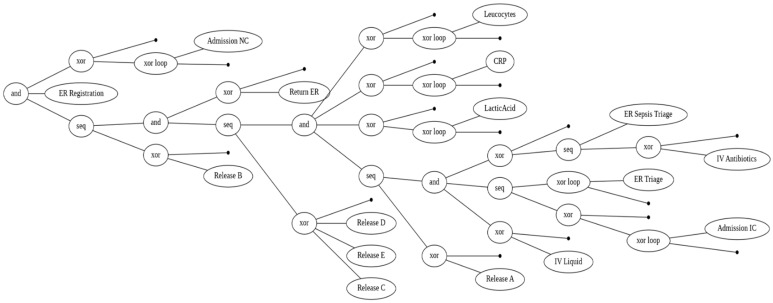
Inductive Miner Process Tree.

**Figure 3:**
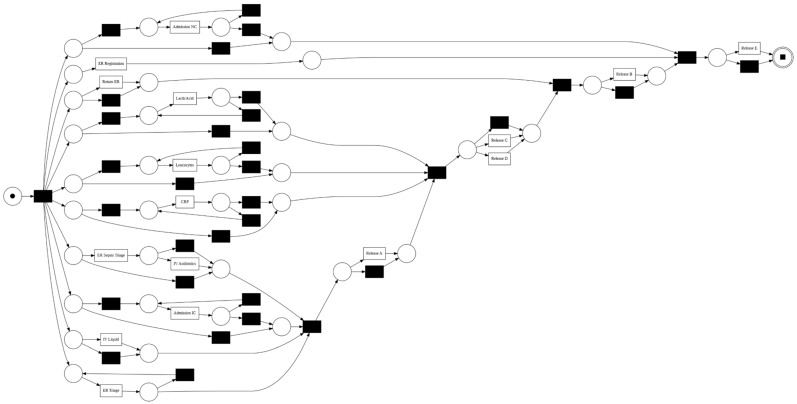
Petri Net model of the discovered sepsis care pathway. Circles represent places, squares represent transitions (activities), and directed arcs denote the flow relations between them.

Figure 2 and Figure 3 present the process models discovered from the sepsis event log using the Inductive Miner. These models provide an overview of the typical clinical pathways observed within the dataset and form the basis for subsequent conformance analysis.

The process tree in Figure 2 offers a hierarchical view of the control-flow structure. It highlights the key sequencing of activities beginning with ER Registration and branching into parallel and alternative behavior. The tree reveals several diagnostic and treatment loops, such as repeated measurements of laboratory tests including CRP, Leucocytes, and LacticAcid. It also shows multiple release transitions (Release A–E), indicating variation in patient discharge destinations.

**Figure 4:**
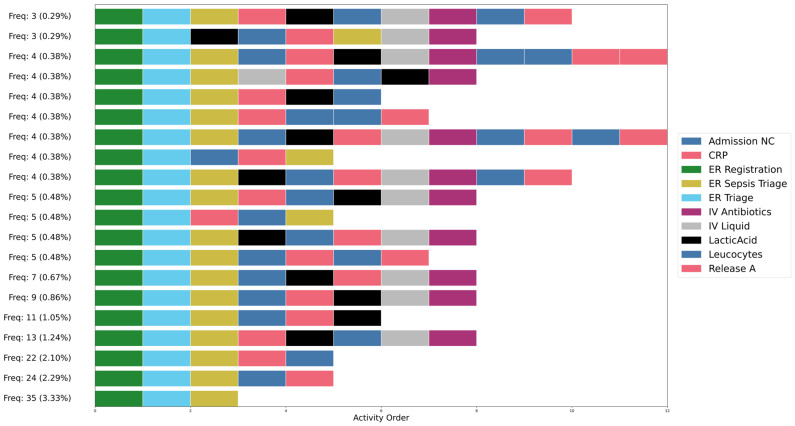
Trace Explorer of Variants with Frequencies.

Figure 3 translates this process tree into an executable process representation. The net visualizes the flow of patients through different clinical stages, from registration and triage to laboratory evaluation, treatment, and eventual discharge or admission. The structure captures both routine and exceptional care pathways, including direct admissions, repeated diagnostic cycles, and return visits to the ER. Figure 4 further presents how patients move through the sepsis care process based on the event log.

Each row of colored blocks shows the activities in the order in which they occurred, and the percentages and frequencies on the left side of each row indicate how often that sequence appears in the dataset. It can be seen that almost all high-frequency variants begin with ER Registration, ER Triage, and ER Sepsis Triage, reflecting the standard entry flow for patients in the emergency department. After these initial steps, the pathways diverge. Some variants proceed with laboratory tests such as CRP, Leucocytes, or LacticAcid, while others include early administration of IV Liquid or IV Antibiotics. The ordering and presence of these steps differ across variants, illustrating variation in diagnostic and treatment decisions.

### 4.2. Comparison of Predictive Models

**Figure 5:**
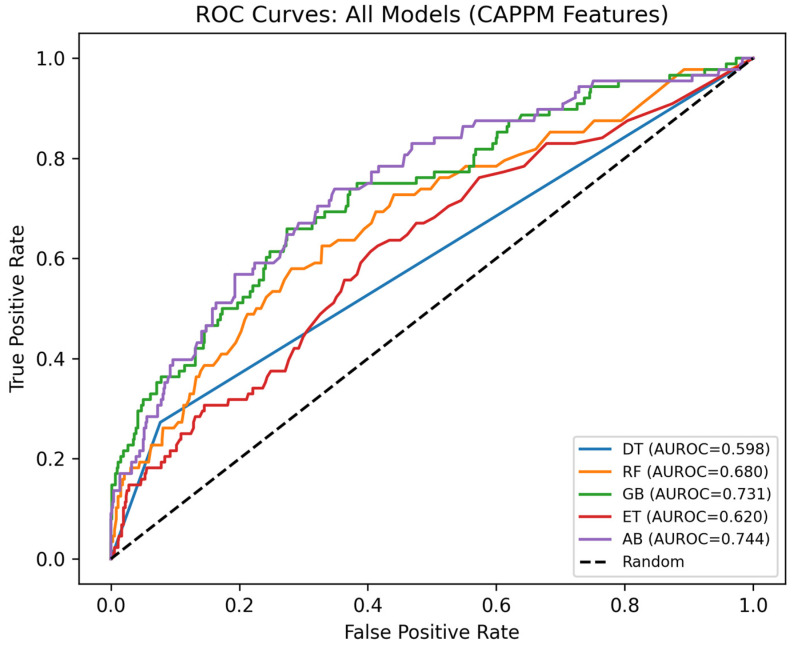
ROC Curves for CAPPM Models.

Figure 5 presents the ROC curves for all models trained using CAPPM features. All models show higher discriminative ability compared to the baseline models, with AUROC values ranging from 0.598 to 0.744, indicating that the CAPPM features effectively separate deterioration from non-deterioration cases. Among the models, the AdaBoost model achieved the highest AUROC of 0.744, closely followed by GB with 0.731 and RF with 0.680. The DT model, however, achieved the lowest AUROC of 0.598. This is because decision trees, unlike ensemble models, are single-split, greedy learners that partition the feature space along one boundary at a time, making them inherently limited in their ability to capture the complex, non-linear interactions between conformance trajectory features, pathway structure, and clinical measurements that characterize sepsis deterioration.

**Figure 6:**
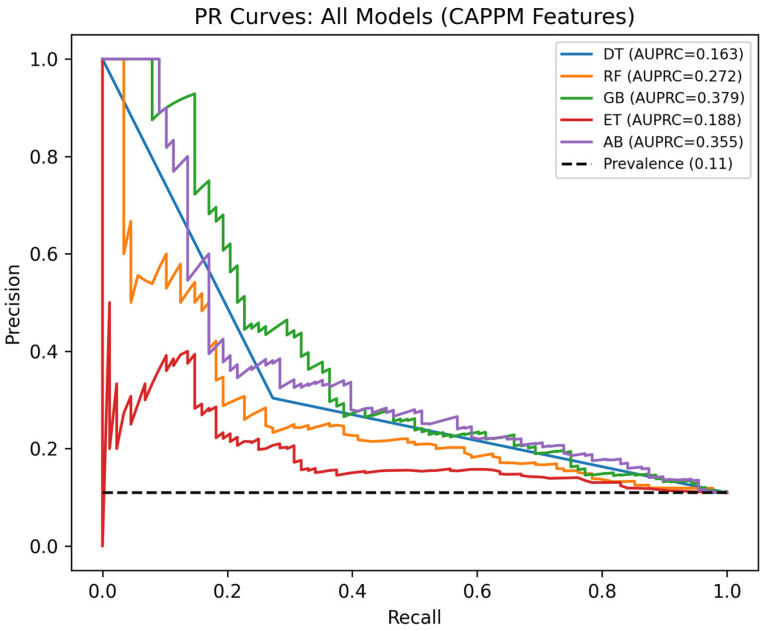
Precision-Recall Curves for CAPPM Models.

Given the class imbalance in deterioration prediction, with only 12.3% of cases representing deterioration outcomes, the Precision–Recall (PR) curves present a more informative assessment of model performance. Figure 6 shows the PR curves for all five classifiers trained on CAPPM features, with the dashed horizontal line indicating the no-skill baseline corresponding to the deterioration prevalence of 0.11 in the test set. All models substantially exceed this baseline across the full recall range, indicating that CAPPM features concentrate true positive cases more effectively than random classification, thus providing a meaningful discriminative signal under real-world class-imbalance conditions.

GB achieved the highest AUPRC of 0.379, followed closely by AdaBoost at 0.355 and RF at 0.272. Extra Trees achieved an AUPRC of 0.188, while the DT yielded the lowest AUPRC of 0.163, consistent with its reduced ability to capture complex nonlinear interactions. However, the absolute AUPRC values remain moderate. This reflects the inherent difficulty of detecting early deterioration using partial trajectory information, where strong clinical signals may not yet be fully expressed. The PR curves further show a progressive decline in precision as recall increases. Capturing a larger proportion of deterioration cases, therefore, requires accepting a higher rate of false positives. This trade-off is expected in imbalanced clinical prediction settings and highlights the importance of selecting an operating threshold aligned with clinical objectives.

**Figure 7:**
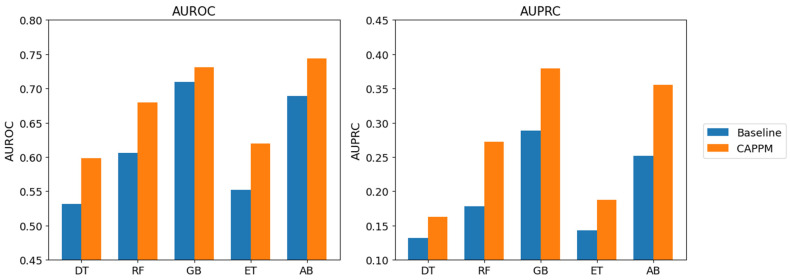
AUROC and AUPRC comparison for Baseline and CAPPM feature sets.

The comparison between the baseline feature set and the full CAPPM feature set in Figure 7 shows consistent improvements in AUROC and AUPRC across all models, confirming that the addition of process conformance, pathway clustering, temporal trend, and DECLARE constraint features systematically enhances predictive performance beyond what static clinical and event-level attributes alone can achieve.

All CAPPM-based models outperform their baseline counterparts, which rely only on static and event-level attributes, indicating improved identification of deterioration cases under class imbalance. For example, GB improves from an AUROC of 0.710 in the baseline setting to 0.731 with CAPPM features, while its AUPRC increases from 0.289 to 0.379. AdaBoost shows a similar pattern, with AUROC rising from 0.689 to 0.744 and AUPRC from 0.294 to 0.355. Random Forest also demonstrates improvement in both metrics. This pattern shows that combining conformance, process, declare, and trend features enables the models to more reliably recognize early deterioration signals. Overall, the figure demonstrates that integrating PM concepts into predictive modelling enhances early sepsis detection performance.

**Figure 8:**
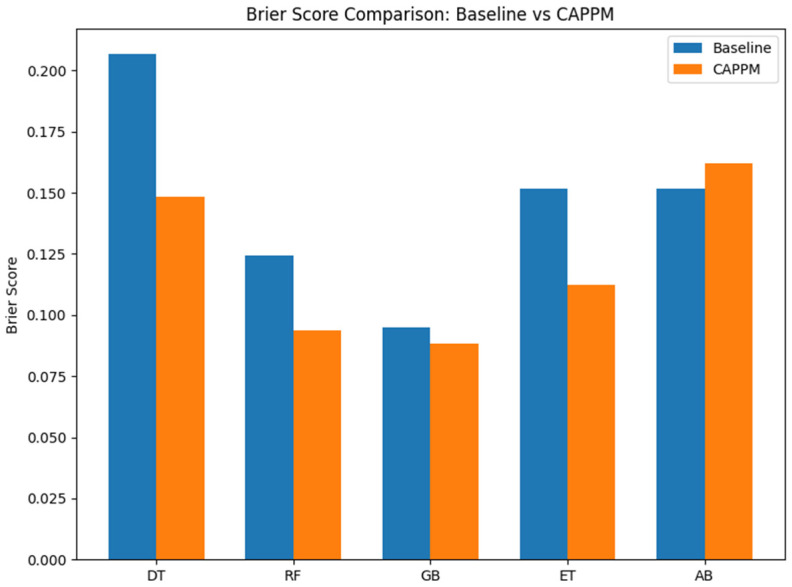
Brier Score comparison for Baseline and CAPPM model.

Figure 8 presents the Brier scores for all classifiers, which measure the mean squared difference between predicted probabilities and observed outcomes, with lower values indicating better probabilistic accuracy and calibration. Four of the five models, incorporating CAPPM features, reduced the Brier score relative to the baseline models. DT improved from 0.207 to 0.148, RF from 0.124 to 0.094, GB from 0.095 to 0.088, and Extra Trees from 0.152 to 0.112. These reductions indicate that CAPPM features not only enhance discrimination but also improve the reliability of predicted probabilities. The most notable improvement is observed for the DT and Extra Trees models, suggesting that process and conformance-based features provide meaningful probabilistic refinement even for less complex learners.

GB with CAPPM features achieved the lowest Brier score overall at 0.088, indicating the most accurate probability estimates among the evaluated models. In contrast, AdaBoost exhibited a slight increase in Brier score when CAPPM features were added, rising from 0.152 to 0.162. Although AdaBoost achieved a high AUROC performance, this result suggests that its probability estimates are less well calibrated compared to GB.

**Figure 9:**
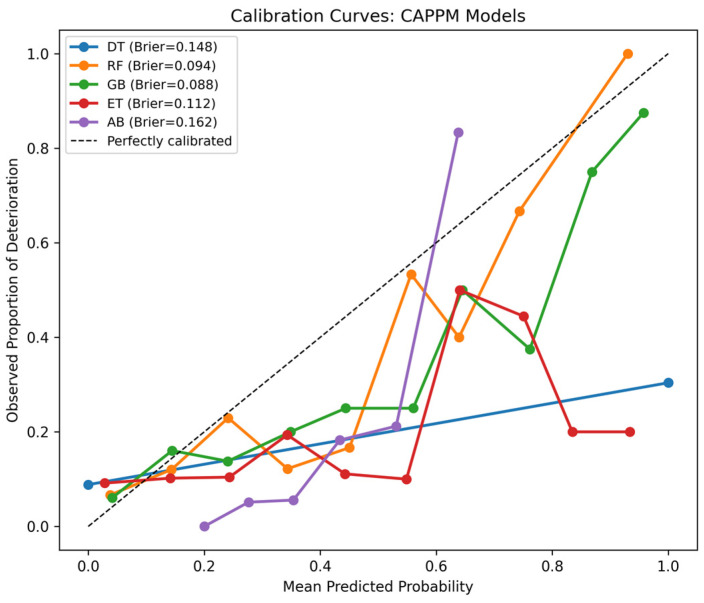
Calibration Curves for CAPPM Models.

Figure 9 further reinforces the calibration performance of the GB model, where it showed near-monotonic alignment with the diagonal reference line across most probability bins, indicating reasonable agreement between predicted risk and observed outcome frequency. Minor deviations were observed in the higher probability ranges, where predicted probabilities slightly underestimated the true event rate. This behavior is consistent with the limited number of cases assigned to high-risk bins in an imbalanced dataset, which increases variability in empirical estimates.

**Figure 10:**
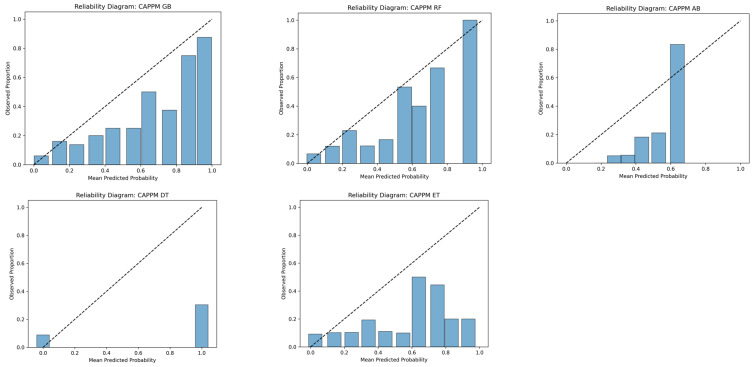
Reliability Diagram for CAPPM Gradient Boosting.

The reliability diagram for the CAPPM models presented in Figure 10 illustrates this relationship by comparing the mean predicted probability within each bin to the corresponding observed proportion of deterioration. The GB model demonstrates acceptable calibration across low- and mid-risk strata, with modest divergence in the upper probability bins. Importantly, no systematic overestimation of risk was observed across the full probability spectrum. Overall, the ensemble-based models, particularly GB and RF, provide not only improved discrimination but also stable and clinically interpretable probability estimates.

**Figure 11:**
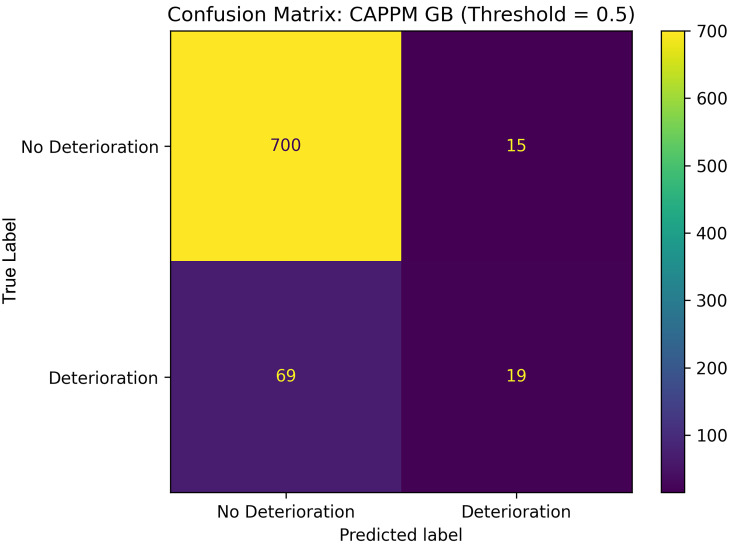
Confusion Matrix for CAPPM GB Model.

The confusion matrix presented in Figure 11 provides a detailed view of the CAPPM GB model’s classification performance at a threshold of 0.5. Among 715 true non-deterioration cases, the model correctly classified 700 and misclassified 15 as deterioration, indicating a low false-positive rate. For deterioration cases, 19 out of 88 were correctly identified, while 69 were misclassified as non-deterioration. This corresponds to a sensitivity of approximately 0.22 at the default threshold. Specificity remains high at approximately 0.98. In other words, the model prioritizes precision and specificity over sensitivity when operating at 0.5, thereby minimizing false alarms but failing to detect a substantial portion. The relatively low number of false positives demonstrates that the CAPPM feature set supports stable discrimination of non-deterioration trajectories. At the same time, the modest sensitivity indicates that a lower probability threshold may be required in practice if the objective is to maximize early detection. Overall, the result indicates that the CAPPM feature set enables the model to make accurate predictions while keeping false alarms minimal, which is important for supporting early sepsis clinical decision-making.

The temporal distance between evaluated prefixes and the first deterioration-defining event was quantified to assess the effective early warning window. Among deterioration cases, the median time between the final evaluated prefix and ICU admission or severe discharge was 3.56 h, with an interquartile range of 1.89 to 21.67 h. These results indicate that risk predictions were generated several hours prior to clinical escalation in a substantial proportion of cases. Notably, one quarter of deterioration cases had more than 21 h between prediction and ICU transfer, demonstrating that the framework captures deterioration risk well before terminal events occur.

### 4.3. Feature Importance and Interpretability

**Figure 12:**
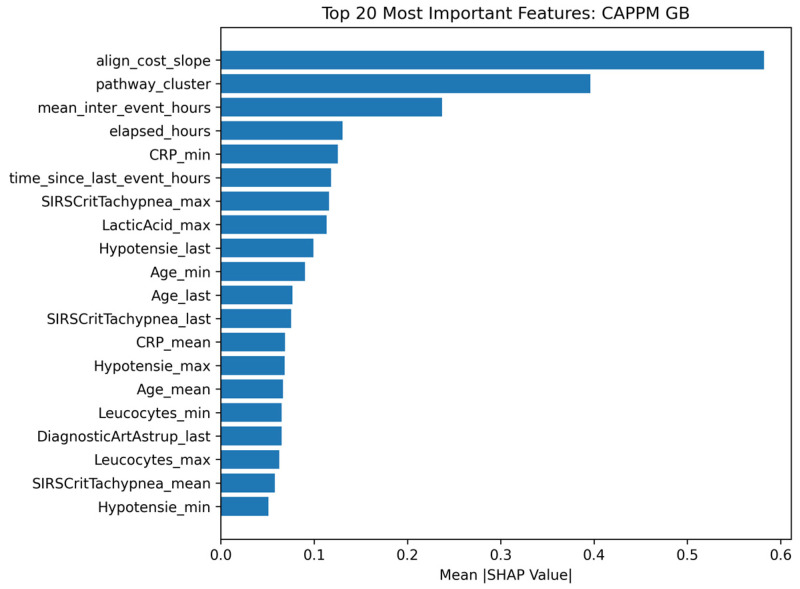
Top 20 most important CAPPM features using SHAP.

The SHAP analysis, presented in Figure 12, highlights the features that contribute most to the prediction of early sepsis deterioration. All SHAP analyses were computed using models trained strictly on prefix-level features, ensuring that feature contributions reflect information available at prediction time. The most influential feature is align_cost_slope, indicating that the rate of change in alignment cost over successive prefixes plays a central role in prediction. A larger slope reflects increasing deviation from the reference process model as the case unfolds. Its dominant contribution suggests that dynamic divergence from expected care pathways is strongly associated with deterioration risk. The second most important feature is pathway_cluster, which captures structural similarities among patient trajectories. Its high importance indicates that pathway-level grouping contains substantial predictive information. This suggests that certain structural care patterns are consistently associated with higher or lower risk profiles.

Temporal process characteristics also contribute meaningfully. mean_inter_event_hours and elapsed_hours appear among the top-ranked features. These variables reflect the pace and progression of clinical activity. Their prominence indicates that timing dynamics, not only event presence, influence predicted risk. Several laboratory-derived features follow in importance, including CRP_min, Leucocytes_min, and CRP_mean, along with physiological indicators such as SIRSCritTachypnea_max, LacticAcid_max, and Hypotensie_last. These features represent early inflammatory and hemodynamic markers. Although their individual contributions are smaller than the leading process-based features, they collectively form a substantial portion of the predictive signal. The distribution of feature contributions supports the CAPPM framework, in which temporal and conformance-based signals constitute primary indicators of early deterioration risk.

The detected process deviations are not only descriptive but may support actionable clinical insights. For example, increasing alignment costs or protocol violations may correspond to delays in treatment administration, missing diagnostic steps, or unexpected workflow transitions. Such deviations can serve as early warning indicators that prompt clinicians to reassess patient management, verify protocol adherence, or accelerate pending interventions. Therefore, CAPPM provides both predictive risk estimation and interpretable workflow signals that may support timely clinical decision-making.

Overall, the results obtained from the analysis indicate that incorporating conformance and pathway-based features into predictive models can serve as an early warning decision support tool in the emergency and acute care environment, where timely intervention is crucial. Unlike traditional risk scores that rely solely on physiological measurements and laboratory values [38], the CAPPM framework can detect early deviations from expected clinical workflows, such as delayed treatments, abnormal workflow sequences, or missing diagnostic steps, by explicitly modeling how care is delivered and how patient trajectories evolve over time before severe deterioration becomes evident. This creates an opportunity for earlier intervention, potentially reducing the likelihood of progression to septic shock, ICU admission, or mortality. Furthermore, with the integration of SHAP, interpretability has been improved as clinicians can relate elevated risk scores to concrete workflow issues rather than unclear model outputs. CAPPM can serve as a practical foundation for more reliable, transparent, and continuously improving sepsis care.

## 5. Limitations and Future Research Directions

### 5.1. Limitations of the Study

The methodology used in this study has several limitations that should be acknowledged. The Sepsis Event Log represents a single-center dataset reflecting Dutch clinical workflows. Therefore, the discovered reference model and pathway structures are institution-specific and may not generalize directly to other healthcare systems without recalibration. In addition, the dataset reflects workflows from a Dutch academic hospital collected several years ago, and clinical practices may differ across regions or evolve over time, which may further limit direct generalization. The size of the dataset limits the ability to evaluate certain models or investigate rare patterns of sepsis deterioration in detail. The study also focused on offline prediction, and the framework has not yet been tested in a real-time environment where data arrive continuously, and decisions must be made under time constraints. Another limitation is the inability to directly compare the proposed framework with established clinical risk scores such as Sequential Organ Failure Assessment (SOFA), Quick SOFA (qSOFA), or National Early Warning Score (NEWS). The event log used in this study primarily contains process and laboratory event information and does not consistently provide the physiological measurements required to compute these scores, including blood pressure, respiratory rate, Glasgow Coma Scale, platelet count, bilirubin, and creatinine. In addition, the dataset lacks consistent demographic and clinical background variables, such as comorbidities or immune status, which are important factors influencing deterioration risk, particularly among elderly, pediatric, or immunocompromised patients. Future work using richer electronic health record datasets may enable benchmarking of CAPPM predictions against standard clinical scoring systems and evaluation of performance across patient subpopulations, supporting development of subgroup-specific predictive models and pathway analyses. Specifically, in a richer dataset, subgroup-specific reference models could be discovered separately for elderly (>65), pediatric, or immunocompromised populations, enabling subgroup-calibrated conformance assessment. These limitations do not reduce the value of the findings but highlight areas where further validation is needed.

### 5.2. Future Research Directions

To address these limitations and strengthen the proposed framework, future research can focus on the following areas:

- Validation across multiple hospitals: Evaluating the framework using event logs from different institutions would help determine how variations in clinical practice affect process behavior and predictive performance.
- Development of real-time monitoring capabilities: The framework can be extended to operate with streaming data so that predictions and conformance insights update as new events occur during patient care.
- Use of adaptive or personalized reference models: Future studies can explore models that adjust to changes in clinical practice over time or that generate patient-specific pathway baselines for improved conformance assessment.
- Integration of federated learning with process mining: This direction would allow hospitals to collaborate on predictive modelling while keeping patient data local, which supports privacy-preserving analytics in healthcare systems [39].

## 6. Conclusions

This study proposed a CAPPM framework for early detection of sepsis deterioration using incomplete care pathways. The approach transformed raw event logs into prefix-based representations of patient trajectories, extracted behavioral, temporal, clinical, pathway, and conformance features, and used these features to train several predictive models. AdaBoost achieved the highest AUROC, while GB demonstrated superior calibration and overall probabilistic reliability, and the confusion matrix showed high specificity with low false positives. Although the results obtained show good performance, the study has several limitations that create opportunities for further work. One important direction identified is the integration of federated learning with process mining so that hospitals can develop predictive models and conformance structures without sharing raw patient data [39]. This could support multi-institutional collaboration while preserving privacy. Additional research can also explore richer temporal abstraction methods, patient-specific pathway modelling, and adaptive alignment techniques that update the reference model as clinical practice evolves.

## Figures and Tables

**Table 1:** Comparison of Process Monitoring Approaches.

Aspect	PM	PPM	CAPPM
Primary Goal	Discover and analyze completed process executions	Predict future outcomes of ongoing process instances	Predict future outcomes while simultaneously evaluating deviations from expected clinical workflows
Data Used	Completed event logs	Partial traces (process prefixes) from ongoing cases	Partial traces enriched with conformance information derived from reference process models
Temporal Perspective	Retrospective analysis	Prospective prediction of ongoing cases	Prospective prediction incorporating evolving conformance behavior
Role of Conformance Checking	Used mainly for retrospective evaluation	Typically not included in prediction	Integrated as a core component to quantify workflow deviations during prediction
Clinical Utility	Identifies bottlenecks and workflow inefficiencies	Provides early prediction of clinical or operational outcomes	Supports early risk detection while revealing workflow deviations that may require clinical intervention
Output	Process models and descriptive workflow insights	Predicted future outcomes	Predicted risk outcomes accompanied by interpretable deviation indicators

**Table 2:** Statistics of Activity Occurrences in the Sepsis Event Log.

Activity	Count	Mean	Std	Min	25%	50%	75%	Max
ER Registration	1050.0	1.000000	0.000000	1.0	1.0	1.0	1.0	1.0
Leucocytes	1050.0	3.221905	4.461176	0.0	1.0	2.0	4.0	74.0
CRP	1050.0	3.106667	3.918626	0.0	1.0	2.0	4.0	69.0
LacticAcid	1050.0	1.396190	2.701760	0.0	1.0	1.0	1.0	51.0
ER Triage	1050.0	1.002857	0.053401	1.0	1.0	1.0	1.0	2.0
ER Sepsis Triage	1050.0	0.999048	0.030861	0.0	1.0	1.0	1.0	1.0
IV Liquid	1050.0	0.717143	0.450602	0.0	0.0	1.0	1.0	1.0
IV Antibiotics	1050.0	0.783810	0.411842	0.0	1.0	1.0	1.0	1.0
Admission NC	1050.0	1.125714	0.877320	0.0	1.0	1.0	2.0	5.0
Release A	1050.0	0.639048	0.480506	0.0	0.0	1.0	1.0	1.0
Return ER	1050.0	0.280000	0.449213	0.0	0.0	0.0	1.0	1.0
Admission IC	1050.0	0.111429	0.335340	0.0	0.0	0.0	0.0	2.0
Release B	1050.0	0.053333	0.224804	0.0	0.0	0.0	0.0	1.0
Release E	1050.0	0.005714	0.075413	0.0	0.0	0.0	0.0	1.0
Release C	1050.0	0.023810	0.152528	0.0	0.0	0.0	0.0	1.0
Release D	1050.0	0.022857	0.149519	0.0	0.0	0.0	0.0	1.0

Release A–E correspond to discharge categories defined in the original hospital information system within the Sepsis Event Log dataset. These categories represent different discharge destinations or outcomes recorded by the hospital, although detailed clinical definitions are not available in the public dataset.

## Data Availability

The data presented in this study are openly available in the 4TU.ResearchData repository as the *Sepsis Cases—Event Log* dataset at https://data.4tu.nl/articles/dataset/Sepsis_Cases_-_Event_Log/12707639 (accessed on 1 January 2026) [31].
